# Design and Validation of a Wearable System for Enhanced Monitoring of Lower Limb Lymphedema

**DOI:** 10.1109/JTEHM.2025.3563985

**Published:** 2025-04-24

**Authors:** Sara Bernasconi, Giovanni Maria Oriolo, Giovanni Farina, Andrea Aliverti, Antonella Lomauro

**Affiliations:** Dipartimento di ElettronicaInformazione e Bioingegneria, Politecnico di Milano 20133 Milan Italy; Istituti Clinici Zucchi di Monza 20900 Monza Italy

**Keywords:** Lymphedema, personalized treatment, physical exercise compliance, ROM detection, wearable device

## Abstract

Lymphedema, characterized by limb swelling, is typically treated with Complex Decongestive Therapy (CDT), which includes physical exercise. This study seeks to design and validate a wearable device aimed at enhancing CDT by monitoring patient adherence to prescribed exercises and tracking changes in the range of motion of the affected limbs. A wearable device, constituted by two boards with 2 IMUs, connected by a flexible flat cable, was designed and developed for placement across targeted joints. It communicates wirelessly with PCs, where raw data from IMUs are collected. Through the application of the Madgwick filter, orientation of the units is obtained and finally joints angles are computed. The device was validated through bench testing using an orthopedic goniometer and field testing with an optoelectronic system. The in vivo validation involved 18 volunteers, including 10 healthy individuals and 8 individuals with lymphedema, who performed flexion-extension movements and walked on a treadmill (at speeds of 3 km/h and 5 km/h). Bench testing demonstrated strong correlation and agreement (r^2^=0.999, mean percentage error = -0.51°, standard deviation = 2.00°). Once worn by the participants, the device enabled the measurement of joint angles during flexion-extension exercises (r^2^=0.852, mean percentage error = 1.44°, standard deviation = 11.7°) and the extraction of step counting, step time and toe off during walk at different speeds. The developed wearable device exhibited robust performance in both bench and field testing. This device, designed specifically for lymphedema patients, offers valuable insights into limb function and exercise adherence, potentially improving personalized treatment strategies.

## I. Introduction

Nowadays, wearable devices are widespread and offer intriguing possibilities when employed within the medical domain. With advances in production technology, wearable devices are miniaturized to guarantee the best wearability and the possibility to acquire biomedical signals also outside the clinical setting [1]. Thanks to the advent of wearable technology, one of the most influenced branches of biomedical research is motion analysis, sports performance evaluation, and rehabilitation.

In the rehabilitation context, wearables offer customized options and personalized feedback to monitor patient functionality throughout the day and to provide assistance during specific physical exercise or treatment at different levels.

This technology overcomes the limitations associated with the conventional rehabilitation process, which typically depend on the expertise and experience of specialists. As a result, it enables an objective evaluation of functionalities and the efficacy of treatment. This also helps in determining the treatment plan aimed at ensuring optimal outcomes [2]. Additionally, wearables enhance patient compliance with rehabilitation plans by monitoring daily activities. Different transducing technologies are used with inertial measurement units (IMUs) standing out as one of the most widely employed and accurate options for this purpose [3]. Standard parameters assessed in rehabilitation are the range of motion (ROM) or joint angles, and the mobility of the affected limb with respect to the healthy contralateral (if present).

A novel and valuable application of wearables for motion analysis during treatment and patient follow-up is for lymphedema.

**FIGURE 1.**
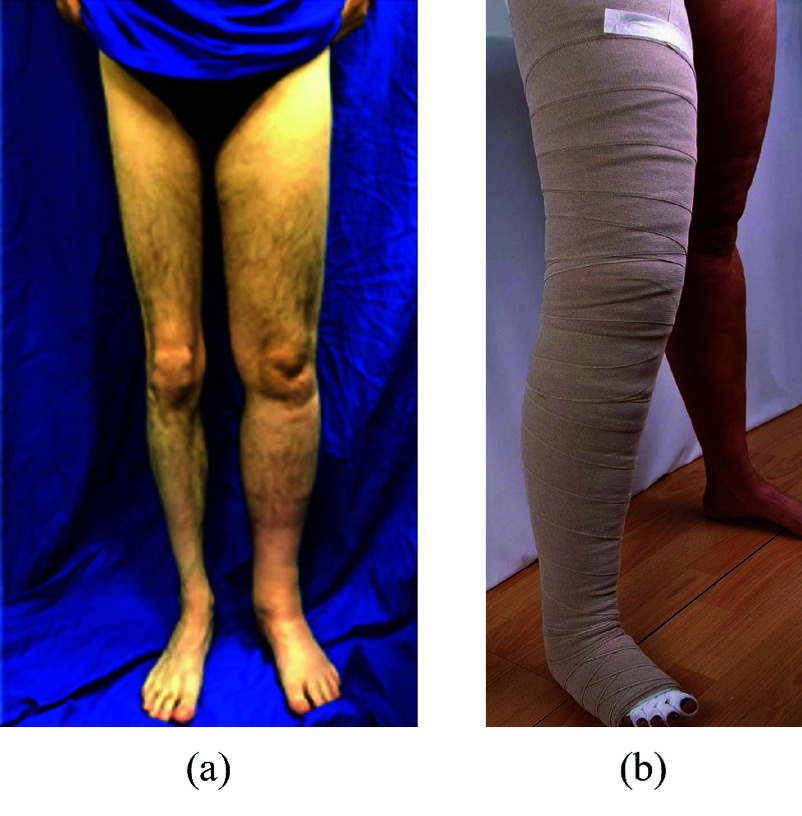
Lower limb lymphedema (a); compression therapy bandaging for the lower limb (b).

Lymphedema is a pathological condition characterized by an accumulation of fluid in the interstitial space due to malfunction of the lymphatic system, causing limb swelling (Figure 1a).

Lymphedema can be primary, a genetic and less prevalent condition, or secondary, generally caused by external factors (typically oncologic surgery or radiotherapy) that damage the lymphatic system. The condition is more prevalent in the lower limbs compared to the upper limbs [4].

There is no cure for lymphedema. The only non-surgical method that helps in containing the swelling of the limbs is the Complex Decongestive Therapy (CDT) [5]. CDT is a multicomponent approach divided into two phases. The first phase is the treatment phase that involves skin care and hygiene, manual lymphatic drainage executed by physiotherapists, multilayer compression therapy and physical exercise. The second phase is the maintenance phase, characterized by daily use of compressive garments.

Compressive therapy is one of the crucial aspects of the treatment. It consists in the application of different layers of short elastic bandaging, as can be observed in Figure 1b, to exert pressures on the affected limb to facilitate the lymph flow, in addition to the muscular pump.

The multilayer compression therapy is done daily (5 days a week) for 2 weeks. To achieve the best outcomes, patients are also advised to perform physical exercises (e.g.: maximal flexion-extension of the joint and walking when the lower limbs are involved) to facilitate lymphatic flow.

However, the swelling induced by lymphoedema can lead to a mechanical impairment in limb’s movement, particularly by restricting the ROM of the affected limb [6]. Indeed, adequate ankle and/or knee dorsiflexion ROM is necessary for daily functional activities such as walking, jogging, and walking up and down stairs [7]. Altered dorsiflexion ROM of the ankle and/or the knee, therefore, can be a risk of chronic biomechanics gait instability [8].

The analysis of the ROM of the joints in lymphedema could play an important role by: 1) quantifying the impairment caused by the condition; 2) determining the effectiveness of the multilayer compression therapy and 3) monitoring functional recovery, as it directly reflects the effects of treatments on joint mobility and lymphatic flow [9], [10], [11], and 4) providing essential insights to tailor rehabilitation strategies.

In this paper, a motion analysis system based on IMUs for lymphedematous patients, which can be embedded in the bandaging has been designed, developed, and validated.

Firstly, a pilot study involving five patients affected by lymphedema at the lower limb was performed to understand the lymphedematous condition in terms of pathophysiology, expected range of motion and technological characteristics that the device should have. The range of motion (ROM) of the ankle and the knee of these patients was measured using an already commercially affirmed IMU device. Then, based on the limitations found for this application, the new IMU device was designed and developed.

The new device was initially validated through bench testing with an orthopedic goniometer, followed by dynamic validation during field testing, using a motion analysis system as the gold standard. The range of motion (ROM) of the ankle and knee in 18 participants, including 10 healthy individuals and 8 with lymphedema, was simultaneously measured using the newly developed device and the optoelectronic system.

## II. Materials and Methods

### A. Pilot Study

A pilot study was initially conducted to familiarize the team with the condition and understand the most suitable characteristics to assess ROM in patients with lymphedema, using a commercialized system, the MTw Awinda (Movella, Henderson, NV, USA).

Data were collected during a campus organized by “Lymphido”, a volunteer organization dedicated to assisting children (and their families) with congenital lymphedema. The protocol was approved by the Politecnico di Milano Ethical Committee (Parere n. 32/2023) and all volunteers signed informed consent forms before the beginning of data acquisition.

The acquisition protocol included:

• Maximal flexion-extension of the knee (4 repetitions)
• Maximal flexion-extension of the ankle (4 repetitions)
• 10 steps walking sequence

The lymphedematous limb was assessed both with and without bandaging, using the healthy contralateral limb as a reference. In cases of bilateral lymphedema, the less affected limb served as the “healthy” reference. This approach allowed for the evaluation of motion impairment due to lymphedema and the effects of bandaging.

Following the company’s guidelines, five MTw Awinda units were placed on specific body parts: one on the back at torso height, one at pelvis height, one on the lateral external side of the thigh, one on the shin, and one on the dorsum of the foot. While no definitive conclusions could be drawn from the campus data, this experience was crucial for identifying the essential characteristics the new device should possess.

For instance, since the device is intended for daily use, minimizing its size was imperative to ensure it could be comfortably worn, even with footwear, and ideally integrated into bandaging. Given the inherent bulkiness of bandaging, a device capable of being seamlessly incorporated into it would be highly desirable.

Moreover, the commercial monitoring system used in the study exhibited interference issues with ferromagnetic elements, such as treadmills. This is incompatible with the intended application of tracking users both indoors and outdoors, where interference cannot be controlled. Therefore, reducing power consumption to enable 24-hour autonomous operation became a priority.

Additionally, the significant variability in morphology and tissue consistency among participants with lymphedema underscored the need for a customizable device.

### B. Design of the New Wearable Device

**FIGURE 2.**
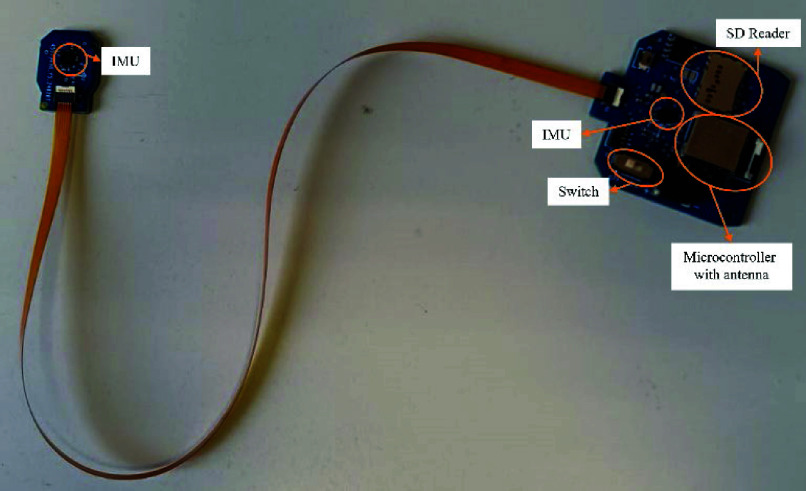
New wearable device, composed by a Main Board (on the right) and a Small Board (on the left), connected by a flat flexible cable (FFC).

The newly designed device, shown in Figure 2, consists of two Printed Circuit Boards (PCBs), with two identical IMUs, connected by a Flexible Flat Cable (FFC). Three FFCs were designed with three standard lengths of 30, 50, and 90 mm to better fit the anatomy of the participants.

The larger board, referred to as the Main Board from now on, has dimensions of $36\times 33$ mm and includes a microcontroller, the nrf52840 (Nordic Semiconductor, Oslo, Norway), one IMU, and other components for the user interface with the device and for conditioning.

The microcontroller communicates via the FFC with the smaller unit, referred to as the Small Board from now on, which measures $13\times 18$ mm and contains only the IMU and some conditioning components. The microcontroller communicates with the 2 IMUs on both boards using I2C, collecting data from both units.

The Main Board is designed to be placed on the proximal body segment of the joint, while the Small Board must be placed in the distal body segment.

The choice of the IMU was driven by considerations of achieving the lowest power consumption possible and the highest accuracy. The chosen model is the 3-axis accelerometer and 3-axis gyroscope LSM6DSO (STMicroelectronics, Geneve, Switzerland) as it provides the best characteristics with low power consumption. In fact, in normal power mode, it consumes only 0.51 mA, reaching values in the microampere ( $\mu $A) range in low power mode. The chosen sensitivities are ±8 g and ±1000 dpi for the accelerometer and gyroscope, respectively.

Additionally, the data compression feature and the FIFO writing procedures offered by the sensor are implemented in the system to further reduce power consumption. The LSM6DSO also has a programmable finite state machine, which can be useful for future implementation of Human Activity Recognition (HAR) or to lighten the computational load on the microcontroller. The IMU was chosen with 6 degrees of freedom instead of 9 degrees of freedom, excluding the magnetometer, to avoid electromagnetic interference.

To facilitate real-time data collection, a sampling rate of 52 Hz was chosen, balancing the need for sufficient temporal resolution with the constraints imposed by the wireless communication protocol.

The wireless communication was conducted using the ANT protocol, a wireless personal network protocol by Garmin Canada, which is well-suited for applications of this kind. The ANT protocol allows for the direct transmission of raw acceleration and gyroscope data from the IMUs to the PC in real-time. To ensure an efficient data transmission, the burst transmission mode was implemented. In this setup, data from the IMUs are first collected in an internal buffer within the microcontroller, then transmitted to an ANT dongle connected to the PC. The transmitted data is then saved in a log file using AntWareII, the dedicated application designed to receive data from the ANT unit.

During the implementation of the wireless communication, it was noted that higher Output Data Rates (ODRs) such as 104 Hz, while potentially providing better accuracy, would lead to higher power consumption and strain the data transmission capacity. Furthermore, the continuous transmission of data streams via the ANT protocol presented intrinsic limitations, prompting the decision to lower the sampling rate to 52 Hz. This sampling rate was selected as a suitable compromise between maintaining sufficient temporal resolution and ensuring reliable data transmission over the wireless connection. The designed device also includes a socket for inserting a microSD card to register IMUs data offline. The SD card writing procedure leverages embedded features of the LSM6DSO. These features help reduce the power consumption of the procedure and manage the time between data collection and writing. IMU data are first recorded in their internal FIFO memory. When this memory is full, the microcontroller reads both units data and begins writing to the SD card. Since the writing procedure takes longer than FIFO readings, a compression algorithm of the IMU was also implemented to store only significant data in the FIFO.

The compression algorithm is particularly useful when the IMU data shows little variability over time, as it allows for the storage of significant data only, reducing the power consumption involved in the SD card writing process. This approach was chosen because the device is primarily intended for real-world use, where offline data storage is crucial for long-term monitoring. It enables the device to store large amounts of data efficiently while minimizing energy consumption, especially when the data is not actively being transmitted. However, for real-time data transmission, such as during specific exercise sessions or specialist visits, we opted to transmit raw data without compression. While compression can offer advantages in memory and power efficiency, it also introduces several drawbacks. First, compression can reduce the accuracy of the transmitted data, as it may lead to a loss of temporal details and lower resolution. When the difference between consecutive sensor readings is minimal, the compression algorithm stores multiple samples in a single FIFO word, which may lead to a loss of fine-grained information. Furthermore, the added complexity of decoding compressed data in real time could introduce delays or errors, which would hinder the accuracy of real-time analysis. Therefore, raw data transmission was prioritized to ensure the highest possible resolution and avoid any potential information loss during critical real-time assessments.

The chosen IMUs are already factory calibrated, but at least at the first power-on, a specific calibration procedure is suggested. Specifically, Gyroscope calibration is performed by placing the sensor on a stable, non-rotating surface along its three axes, where raw data are averaged over time to determine and correct for offset values. Similarly, accelerometer calibration involves positioning the sensor in six distinct orientations to measure the gravitational acceleration vector. This process enables precise identification and compensation of offsets, thereby ensuring accurate measurements.

From the linear acceleration and angular velocity data, joint angles are derived using the X-io Technologies open-source firmware. Specifically, this algorithm employs the Madgwick filter, a sophisticated sensor fusion technique designed to estimate device orientation by integrating gyroscope and accelerometer measurements [12]. Developed by Sebastian Madgwick, this filter is renowned for its efficiency in providing accurate, real-time orientation estimates with minimal computational demands. The algorithm operates by minimizing the discrepancy between predicted and measured sensor data through a gradient descent optimization method to refine orientation estimations.

The Madgwick filter is particularly advantageous in dynamic conditions, delivering high-quality orientation estimates with low latency, which is critical for precise spatial tracking applications. Initially, the algorithm calculates quaternions, which are more robust against noise, drift, and gimbal lock compared to Euler angles. Subsequently, the quaternions are converted into Euler angles (yaw, roll, and pitch) for each of the two IMUs, thus defining their respective orientations. Finally, the joint angle in the sagittal plane is determined by an algebraic addition of the pitch values from the two IMUs.

### C. Bench Testing

**FIGURE 3.**
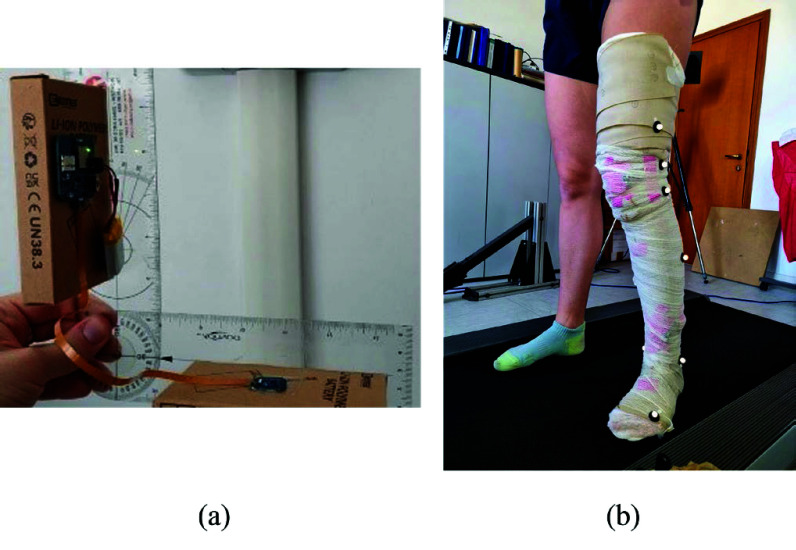
Bench testing with orthopedic goniometer of the new device (a); field validation on one subject. The device was positioned before the last layers of the bandaging. The visible passive markers were spherical and positioned following the Davis protocol.

The new device was validated through bench testing using an orthopedic goniometer. The units were placed on each arm of the goniometer, as shown in Figure 3a. Multiple acquisitions were conducted, testing angles ranging from 0° to 360°. The validation results are presented in terms of correlation line and coefficient, Bland-Altman analysis, and percentage error between the device and the gold standard.

### D. Field Testing

Eighteen participants were enrolled in the experimental trial, approved by Politecnico di Milano Ethical Committee (Parere n.20/2024). The trial comprised 10 healthy individuals (6 males, age 24.5 ± 0.5 years, weight 68.3 ± 12.9 kg, height 175.1 ± 11.1 cm) and 8 individuals with lower limb lymphedema (3 males, age 59.1 ± 14.3 years, weight 73.3 ± 13.3 kg, height 167.9 ± 7.5 cm). All volunteers signed informed consent forms before the beginning of data acquisition.

The gold standard for this study was the optoelectronic motion analysis system (SMART system, BTS Bioengineering, Milan, Italy), which utilizes 8 infrared cameras.

The sampling frequency was set at 100 Hz, and passive spherical markers were placed on the studied limb, following the Davis protocol [13], as shown in Figure 3b.

Two sets of devices were used: one for the knee joint and one for the ankle joint. These were fixed with kinesiological tape under the final layer of bandages.

The validation protocol (similar to that used at the Lymphido campus) included:

• Maximal and non-maximal flexion-extension of the knee
• Maximal and non-maximal flexion-extension of the ankle
• Walking on the treadmill at two speeds: 3km/h and 5 km/h (only for the individuals without lymphedema).

The protocol was repeated twice for each limb: once without bandages and once with bandages.

**FIGURE 4.**
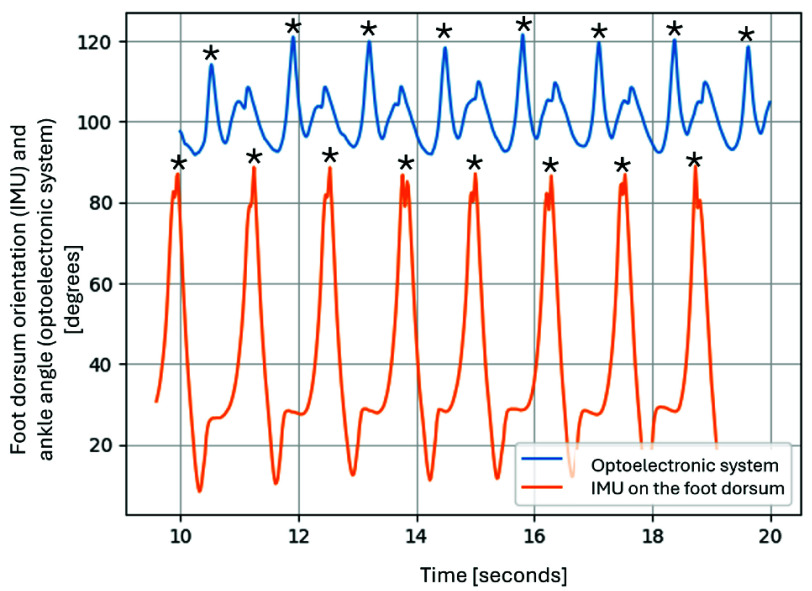
Field testing results for step counting during the 3 km/h walking test. The figure shows ankle angles recorded by the optoelectronic system (blue) and the sagittal plane orientation relative to the ground, calculated from IMU data on the foot dorsum (orange). Black stars mark the points where steps are counted.

The flexion-extension angle of both joints on the sagittal plane was computed. For the walking test, temporal features assessed by the two systems were compared to determine if the developed device could accurately track gait. Specifically, the system’s capability to detect the correct number of steps was evaluated by manually identifying peaks in the optoelectronic signal and comparing them with those from the IMU on the dorsum of the foot. A representative example of the step counting is shown in Figure 4, which illustrates the ankle angle recorded by the optoelectronic system and the orientation of the IMU on the dorsum of the foot. The step time and toe-off detection were also extracted for the two speeds.

### E. Agreement and Accuracy Assessments

To assess the agreement between the newly designed device and the motion analysis system, a correlation analysis was conducted. Linear correlation indices, including slope (m) and intersection (q) of the interpolation line, as well as the coefficient of determination (r^2^), were computed. Additionally, the Bland-Altman plot was generated. These analyses were performed separately for both the bare and banded limb conditions. The accuracy of the device in the non-banded condition versus the banded condition was evaluated using either the t-test or the Mann-Whitney Rank Sum Test, depending on the results of normality tests. The level of significance was set at p<0.05.

The percentage (%) error was also calculated as the absolute difference between the measurements from the newly designed device and the gold standard, divided by the measurements from the gold standard.

## III. Results

### A. Bench and Field Testing Results

For the bench testing, the correlation line between the new device and the goniometer angle presented values of slope of 0.998 and an r^2^ value of 0.999 with an intercept of 0.314°. The mean percentage error of the two systems was −0.51°, calculated as the goniometer reading minus the new device measurement, with a standard deviation error of 2.00°.

**FIGURE 5.**
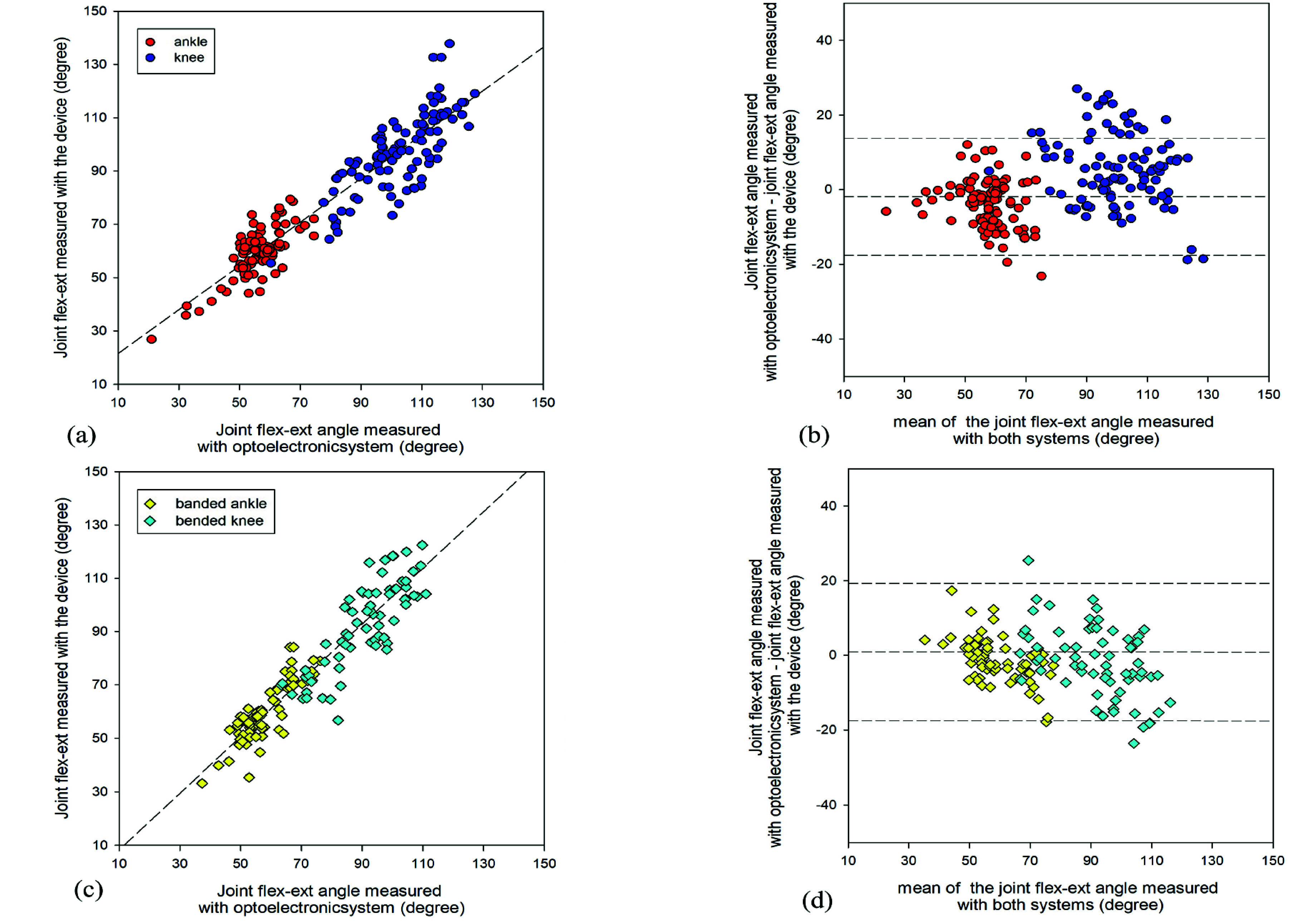
Field validation results of joint angles of the ankle and knee, with and without bandages during flexion extension measured by the new device versus the optoelectronic system. In (a) the correlation line of the bare leg and (b) the Bland-Atman plot, where red and blue dots representing respectively the ankle and knee angles. In (c) and (d) the correlation line and Bland-Altman plot of the banded acquisitions are reported, with yellow and light blue square representing ankle and knee angles, respectively.

For the field measurement, the overall correlation line of the joint angles (knee and ankle) between the two systems showed a slope of 0.878 and an r^2^of 0.852, with an intercept of 9.75°. The corresponding Bland-Altmann analysis of overall data showed a mean difference of -1.44°, considering the optoelectronic measurements minus the new device measurements, with a standard deviation of 11.7°. Correlation and Bland-Altman plot of the bare and banded joints are reported in Figure 5 (a-d).

For the walking acquisitions, the number of steps counted by our device and the optoelectronic system perfectly match. The slope and r^2^ values of the correlation line for the 3 km/h test were 0.922 and 0.899, respectively. The Bland-Altmann analysis of the same trial revealed a mean error of 2 ms and a standard deviation of −84.1 ms, with the values of toe-off being generally more dispersed (greater standard deviation) with respect to the step time.

At 5 km/h, the correlation line presented values of slope and r^2^ values respectively of 0.870 and 0.847. The Bland-Altman shows a mean error of −20 ms and a standard deviation of 87.4 ms. Similarly to the 3 km/h trial, the toe-off detection presented a more widespread result.

### B. Banded Versus Non-Banded Acquisitions

The correlation line for joint angle measurements with the banded limb shows a slope of 1.054, an r^2^ of 0.874, and an intercept of −2.17°, reported in Figure 5a. The corresponding Bland-Altmann analysis reveals a mean difference of −2.32°, calculated as the optoelectronic measurements minus the new device measurements, with a standard deviation of 10.25° (Figure 5b).

For measurements taken with the bare limb, the correlation line shows a slope of 0.820, an r^2^ of 0.873, and an intercept of 13.37°, reported in Figure 5c. The Bland-Altman analysis for these measurements indicates a mean difference of −0.79°, calculated as the optoelectronic measurements minus the new device measurements, with a standard deviation of 11.74° (Figure 5d).

Tables 1 and 2 demonstrate that no significant differences were found between the newly designed device and the motion analysis system regarding flexion-extension angles and temporal features during walking. The new device performed similarly to the gold standard for both joints (ankle and knee) and in banded and non-banded conditions.

**TABLE 1:** Statistical Results of Bare and Banded Ankle and Knee Joints Angle Assessed by the Motion Analysis System and the New Device

Acquisition condition	Joint	Motion Analysis System [°]	New Device [°]	p-value
Bare	Ankle	55.9 ± 7.4	59.1 ± 8.0	0.234
	Knee	102.5 ± 11.4	97.8 ± 12.2	0.264
Banded	Ankle	57.5 ± 7.1	59.0 ± 9.9	0.669
	Knee	90.0 ± 11.0	91.9 ± 15.5	0.683

**TABLE 2:** Step Time and Toe-Off Time of Bare and Banded Condition During Walk Assessed by the Motion Analysis System and the New Device

	Acquisition condition	Walking Speed	Joint	Motion Analysis System [ms]	New Device [ms]	p-value
Step Time	Bare	3 km/h	Ankle	133O ± 50	1300 ± 50	0.234
			Knee	1310 ± 50	1280 ± 40	0.264
		5 km/h	Ankle	1010 ± 60	1010 ± 50	0.669
			Knee	1030 ± 50	1030 ± 60	0.683
	Banded	3 km/h	Ankle	1240 ± 100	1230 ± 70	0.577
			Knee	1290 ± 60	1270 ± 60	0.446
		5 km/h	Ankle	1010 ± 50	1000 ± 60	0.758
			Knee	1010 ± 40	1000 ± 80	0.814
Toe-off Time	Bare	3 km/h	Ankle	820 ± 50	910 ± 50	0.001
			Knee	820 ± 70	770 ± 120	0.303
		5 km/h	Ankle	530 ± 100	64 0 ± 160	0.073
			Knee	640 ± 40	620 ± 60	0.401
	Banded	3 km/h	Ankle	740 ± 120	770 ± 170	0.274
			Knee	790 ± 80	790 ± 170	0.936
		5 km/h	Ankle	570 ± 50	640 ± 110	0.121
			Knee	610 ± 30	630 ± 130	0.589

## IV. Discussion

The newly designed device shows very low dimensions with respect to commercially available devices, especially the small unit, thought to be placed on the foot or, in the case of upper limb lymphedema, on the hand. This compactness is crucial in the first case for wearing it with shoes and in general to enhance comfort. This was achieved thanks to the design, which incorporates a single microcontroller to manage two separate IMUs positioned on different body parts, with the added advantage of requiring only one battery for both units, connected via a Flexible Flat Cable (FFC). The FFC could be tailored to accommodate the individual’s specific length requirements.

In the present study, three different lengths of the FFC were tested. The length of the FFC was determined based on measurements taken during laboratory testing with participants, considering various placements on the knee, ankle, and wrist to explore different configurations. The FFC must be long enough to ensure both units are securely anchored during maximal movements, while also maintaining the correct positioning of the sensors near the joint to allow for accurate representation of the joint angle. Given that FFCs are low-cost and feature an adjustable length design, tailoring the length to the specific needs of each patient could be particularly beneficial, especially for those who need to use the device over extended periods. This system was tested exclusively for lower limbs for practical reasons, as lymphedema is more common in the lower limbs. However, it is also suitable for evaluating upper limbs.

The new device demonstrated good correlation with the gold standard in bench testing, with a correlation line close to the identity line.

In the field validation, the device performed well for the maximal flexion extension of the ankle and the knee, as well as for temporal gait parameters. The mean percentage errors were low for both the analysis done. In the maximal flexion extension, the mean error around 1.5° is considered a good result, while the standard deviation is in accordance with literature for wearable systems based on IMUs [14], [15]. The standard deviation is generally high for flexion extension results. This could be related to the subject dependency of the test evaluated but also to general consideration for IMUs devices such as the unit placement on body segments [3].

The step-counting function of the new device showed perfect agreement with the gold standard. This feature is particularly important for two reasons. Firstly, specialists highly recommend walking as a form of physical activity, as it aids in fluid drainage. These systems can assist in monitoring patients’ adherence to this recommendation. On the other hand, step counting is not so trivial as it seems and other wearable devices, for instance wrist-worn devices, have an error rate of at least 5% or higher [16].

For the temporal gait analysis, both the correlation line and Bland-Altman analysis showed worse results for the 5 km/h, with a mean error of one order of magnitude higher and a wider standard deviation, however acceptable. This can be due to the sample frequency of the system which is not suitable for high-speed motion.

Despite the general limitation related to the sample frequency for high-speed movements, it must be considered that the physiotherapist usually suggests not to perform running while the condition is in acute form.

The comparisons in terms of agreement and correlation with the gold standard in the non-banded and banded condition demonstrate that the position of the IMU as it will be in the real world, (i.e., beneath the last layer of the bandages), show a lower standard deviation. Overall, the results encourage that the device works embedded into the bandaging with similar behavior respect to the bare leg condition.

While the proposed system does not directly guide the placement of bandages, it provides critical data on the ROM and the freedom of movement allowed by the bandage. This information is pivotal for evaluating the effectiveness of compressive therapy in reducing lymphedema and promoting the recovery of full limb mobility. As highlighted in a previous study [17], objective assessments of limb functionality, such as ROM, provide critical insights that help translate research findings into clinical applications. This further reinforces the important role of functional evaluations during the management of lymphedema. Notably, in clinical practice, specialists typically assessed ROM manually. These measurements are therefore operator dependent, and they be less accurate and highly variable and they can introduce potential biases [18]. Therefore, an automated system capable of objectively detecting and computing ROM is highly valuable, ensuring more consistent and reliable evaluations.

Evaluating ROM not only during maximal movements but also during walking enhances the translational impact of our results, as we have considered an important functional activity of daily life.

In addition, the bandages’ impact on the mobility of the joints is thought to lack repeatability, as no standardized tool currently exists to guide professionals or caregivers in applying bandages with consistent pressure while ensuring adequate freedom of movement. A cross-sectional observational study [19] highlights the lack of standardization in the application of compression bandaging, demonstrating that the pressures exerted by bandages can vary significantly. This variability in pressure affects freedom of movement and, consequently, the therapeutic outcome. A practical test conducted with 1,476 participants—divided into those with specific and non-specific expertise—showed that only a small percentage of participants achieved the target pressure range of 50-60 mmHg. Most users, regardless of expertise, were unfamiliar with proper materials or their correct application, resulting in substantial deficits in both knowledge and practical skills.

In the present study, all bandages were applied by the same individual to eliminate variability as a potential confounding factor. However, this can also be considered a limit of the study due to the operator’s dependency on the bandage and the lack of bandage standardization.

Future studies involving multiple professionals and caregivers could demonstrate how systems like the proposed device could aid in standardizing and optimizing the bandaging process, ensuring better consistency and therapeutic outcomes.

The device was very accurate in assessing the temporal features during walking. However, another limitation was the manual evaluation of the system’s capability to detect the correct number of steps. An important future development would be the automatic detection of temporal features and ultimately activity recognition. The development of robust algorithms is acknowledged as a challenge and is currently under investigation. The goal is to either select an existing algorithm capable of handling the typical variations seen in lymphedema patients or train a neural network using a sufficiently large dataset tailored to the unique gait characteristics of this pathology.

Integrating more than two IMUs into a single device could improve motion detection by enabling better synchronization and providing a more detailed assessment of overall body movement. However, the decision to use only two IMUs per Main-Small board pair was guided by practical considerations. Adding additional IMUs to a single Main board would increase power consumption, requiring larger battery capacities. This would result in bulkier and heavier devices, which could cause discomfort or even pressure sores when worn under compression bandages for 24 hours, particularly on the fragile lymphoedematous skin.

Moreover, lymphedema typically affects a single limb or joint (e.g., the ankle), and bandages are often applied only to the affected region. A modular design, as proposed in this study—with each device tailored to a specific joint—is therefore more suitable for the intended application.

In scenarios where multiple joints (e.g., the knee and ankle) need to be monitored, using separate devices on the same body segment (e.g., the lower leg) offers an additional benefit. This configuration provides redundancy in orientation data for that segment, which could be exploited in future developments to further enhance the system’s performance.

## V. Conclusion

Lymphedema is characterized by pathological limb swelling, for which there is currently no cure. However, therapy focuses on managing swelling through specialized drainage massages, applying compressive bandages, and recommending specific physical exercises such as flexion extensions, heel raises, and walking. While specialist interventions address the primary aspects of care, patients are responsible for engaging in physical exercise. Therefore, a wearable device capable of tracking physical activity and thus assessing therapy adherence is valuable. Furthermore, due to the intrinsic characteristics of the pathology to be highly patients specific in terms of morphology and tissue consistency, a wearable device, offering insights into the affected limb’s range of motion, could serve as a crucial tool for tailoring therapy to optimize results.

A new device, based on IMUs, to be inserted under the bandages to study motion of lymphedematous patients has been designed and validated, and, to the best of the authors knowledge, it is the first device specifically designed for this purpose.

The device demonstrated excellent results in both bench testing (r^2^ = 0.999), and field testing (r^2^=0.852 for joints’ angle acquisition, r^2^=0.899 for walking at 3 km/h, and r^2^=0.870 for 5 km/h walking).

It also performed well under bandages, making it suitable for clinical applications. The inclusion of an SD card socket allows for offline and long-term testing, both indoors and outdoors.

In addition, the device provides good results during the walking trails with possible future implementation in terms of Human Activity Recognition which is crucial for rehabilitation programs.

Future research should focus on improving the sampling frequency and testing the system for upper limb lymphedema and longer acquisition periods.

## Conflicts of Interest

The authors declare that there are no conflicts of interest regarding the publication of this paper.

## Author Contributions

**Sara Bernasconi**: Conceptualization, Data curation, Formal Analysis, Methodology, Visualization, Investigation, Writing-original draft, and Writing-Review and Editing. **Giovanni Maria Oriolo**: Methodology, Software, Validation, Formal Analysis, Investigation, and Data Curation. **Giovanni Farina**: Conceptualization, Validation, Investigation, and Resources. **Andrea Aliverti**: Methodology, Resources, Writing-original draft, and Visualization. **Antonella LoMauro:** Conceptualization, Methodology, Validation, Formal analysis, Investigation, Resources, Writing-Original Draft, Writing – Review and Editing, Visualization, Supervision, and Project administrator.
